# Supplementary material for: Light-driven biological actuators to probe the rheology of 3D microtissues
Source: Nat Commun. 2023 Feb 9;14:717. doi: 10.1038/s41467-023-36371-w (PMC9911700; doi:10.1038/s41467-023-36371-w)
Supplement: Supplementary file 1 — Supplementary Information [file 41467_2023_36371_MOESM1_ESM.pdf]

Light-driven biological actuators to probe the rheology of 3D microtissues

Supplementary Information

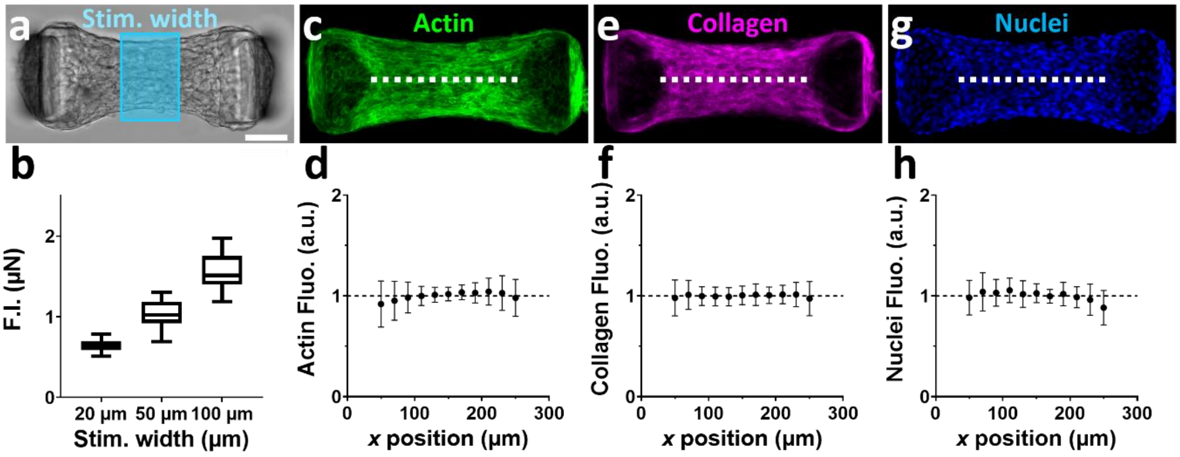

Supp. Figure 1. Light-induced contraction is proportional to the number of stimulated cells. Representative microtissue with the stimulation pattern in blue (a) and corresponding force increase (F.I.) in function of the stimulation width (b). Data are presented as Tukey box plots (i.e. the box extends from the 25th to 75th percentiles, the median is plotted as a line inside the box and the whiskers extend to the most extreme data point that is no more than 1.5 times the interquartile range from the edge of the box) with n = 20 microtissues over 2 independent experiments. Confocal projections and average fluorescence intensity along the dotted line for microtissues stained for actin (c, d), collagen (e, f) and nuclei (g, h). Data in d, f and h are presented as mean ± SD with n = 20 microtissues over 3 independent experiments. Scale bar is 100 µm. Source data are provided as a Source Data file.

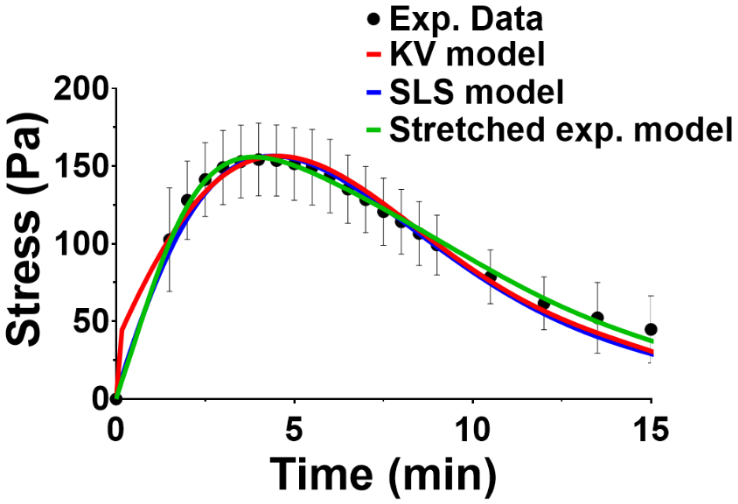

Supp. Figure 2. Viscoelastic modeling, based on [1], of stress response to a light stimulation at t = 0. The black dots correspond to the experimental data obtained in Figure 4 for a stimulation width of 50 µm, and presented as mean ± SD with n = 20 microtissues over 2 independent experiments. The red curve corresponds to the Kelvin-Voigt (KV) model (a spring and a dashpot in parallel), the blue curve to the standard linear solid (SLS) model (a spring and a dashpot in series, in parallel to another spring), and the green curve to the stretched exponential model (a sum of Maxwell bodies, i.e. a spring and a dashpot in series, with a specific distribution of time constants). The corresponding equations and parameters are the following:

- **KV model:** $\boldsymbol{\sigma_{xx} = E.\varepsilon_{xx} + \eta\dot{\varepsilon}_{xx}}$ with E = 18.7 kPa and η = 1.9 MPa.s, leading to a retardation time $\tau = \frac{\eta}{E}$ = 101 s.
- **SLS model:** $\boldsymbol{\sigma_{xx} = \varepsilon_{xx}\left(E_1.e^{-\frac{t}{\tau}} + E_2\right)}$ with E_1 = 56.3 kPa, E_2 = 14.7 kPa and τ = 135 s.
- **Stretched exp. Model** [1]: $\boldsymbol{\sigma_{xx} = \varepsilon_{xx}\left(E_1.e^{-\left(\frac{t}{\tau}\right)^{\beta}} + E_2\right)}$ with E_1 = 65.0 kPa, E_2 = 10.0 kPa, τ = 129 s and β = 0.66

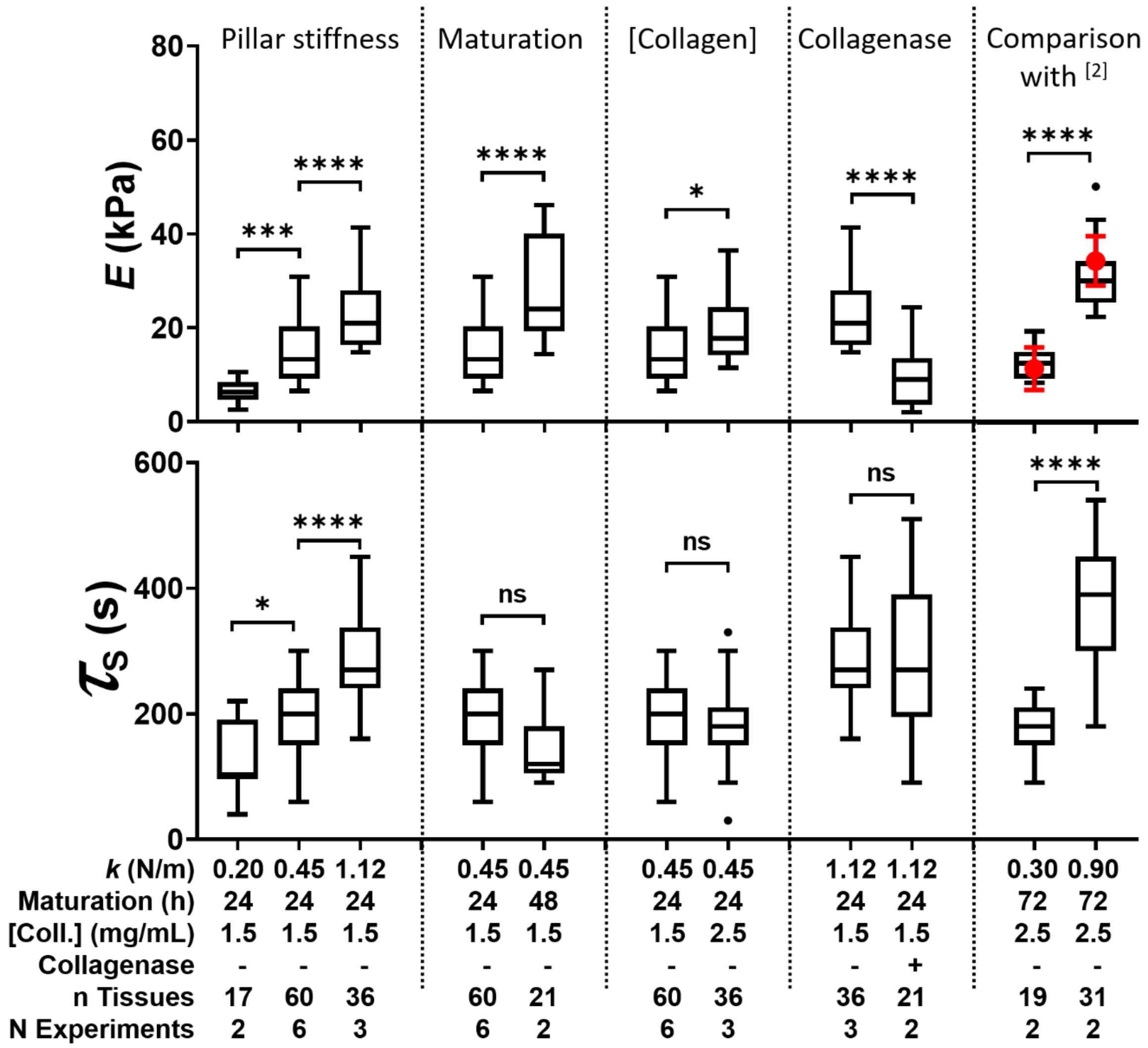

Supp. Figure 3. Optogenetic assessment of the rheological properties of microtissues. Elastic modulus E and delay τ_S between maximum stress and stretch for five different spring constants k of the cantilevers, two durations of maturation, two collagen densities [Coll.] and the presence or absence of collagenase. Data are presented as Tukey box plots (i.e. the box extends from the 25th to 75th percentiles, the median is plotted as a line inside the box and the whiskers extend to the most extreme data point that is no more than 1.5 times the interquartile range from the edge of the box) with n microtissues over N independent experiments (n and N are indicated under the x-axis). The two last columns present mean elastic moduli ± SD obtained with magnetic actuation in [2] (red dots) and with the approach of the present study (box plots) for the same culture conditions. ****P < 0.0001, ***P < 0.001, **P < 0.01, *P < 0.1 and n.s. stands for non-significant (i.e. P > 0.05). Statistical significances were determined by one-way analysis of variance (ANOVA) corrected for multiple comparisons using Tukey test. Source data are provided as a Source Data file.

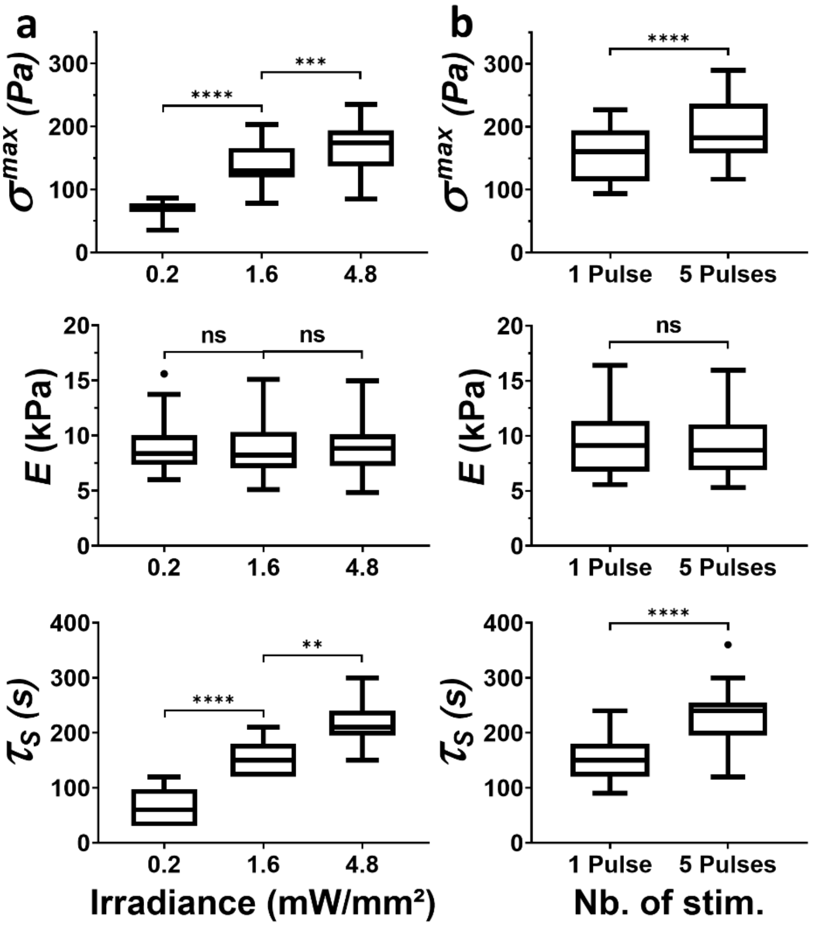

Supp. Figure 4. Influence of the amplitude of contraction on the measured mechanical properties. (a) Light-induced maximum stress σ^{max}, elastic modulus E and delay τ_S between maximum stress and ε_{xx} strain for an irradiance ranging from 0.2 to 4.8 mW/mm². Data are presented as Tukey box plots (i.e. the box extends from the 25th to 75th percentiles, the median is plotted as a line inside the box and the whiskers extend to the most extreme data point that is no more than 1.5 times the interquartile range from the edge of the box) with n = 13 microtissues over 2 independent experiments. ****P < 0.0001, ***P < 0.001, **P < 0.01 and n.s. stands for non-significant (i.e. P > 0.05). Statistical significances were determined by one-way analysis of variance (ANOVA) corrected for multiple comparisons using Tukey test. (b) Light-induced maximum stress σ^{max}, elastic modulus E and delay τ_S between maximum stress and ε_{xx} strain for one pulse or a series of five, 1-minute-spaced pulses of 1.6 mW/mm². Data are presented as Tukey box plots for n = 22 microtissues over 2 independent experiments. ****P < 0.0001 and n.s. stands for non-significant (i.e. P > 0.05). Statistical significances were determined by paired, two-tailed t-tests. Source data are provided as a Source Data file.

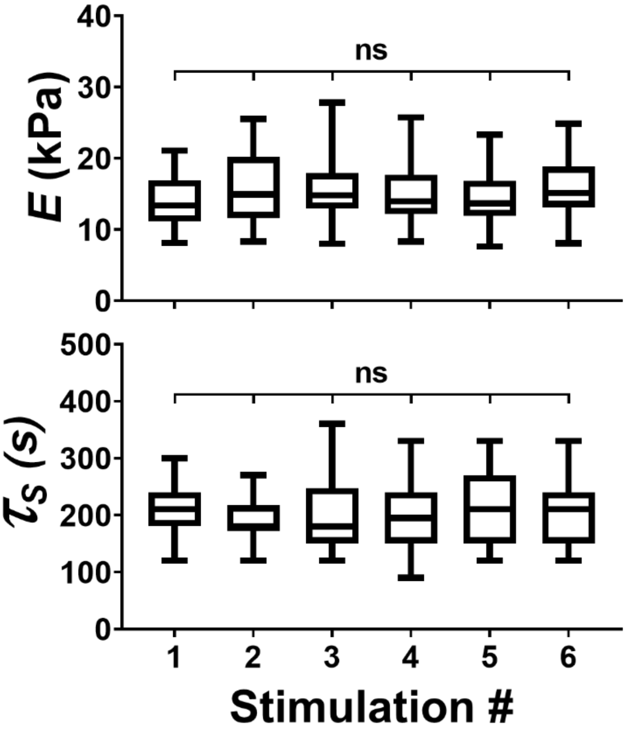

Supp. Figure 5. Optogenetic stimulations do not induce plastic, irreversible deformations of the microtissue. Elastic modulus E and delay τ_S between maximum stress and ε_{xx} stretch for six successive stimulations spaced far enough apart in time to allow complete relaxation (30 minutes between each stimulation). Data are presented as Tukey box plots (i.e. the box extends from the 25th to 75th percentiles, the median is plotted as a line inside the box and the whiskers extend to the most extreme data point that is no more than 1.5 times the interquartile range from the edge of the box) with n = 30 microtissues over 2 independent experiments. n.s. stands for non-significant (i.e. $P > 0.05$). Statistical significances were determined by one-way analysis of variance (ANOVA) corrected for multiple comparisons using Tukey test. Source data are provided as a Source Data file.

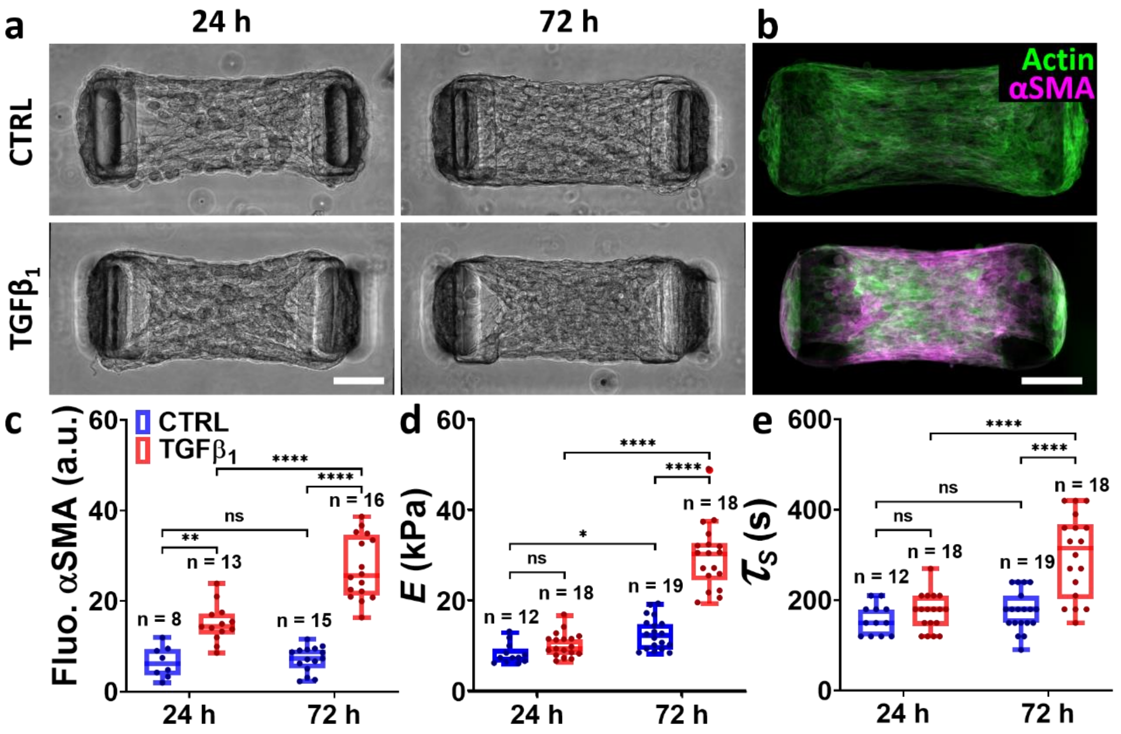

Supp. Figure 6. Myofibroblasts strongly impact the mechanical properties of microtissues. (a) Brightfield images of representative microtissues after 24 h and 72 h of culture in serum-only (CTRL) and TGF-β_1 treated medium (TGFβ_1). (b) Corresponding confocal projections for microtissues stained for actin (green) and α-SMA (magenta) at 72 h. Fluorescence intensity of the α-SMA staining (c), elastic modulus E (d) and delay τ_S between maximum stress and ε_{xx} strain (e) for microtissues after 24 h and 72 h of culture in in serum-only (CTRL, in blue) and TGF-β_1 (in red) treated medium. Data are presented as Tukey box plots (i.e. the box extends from the 25th to 75th percentiles, the median is plotted as a line inside the box and the whiskers extend to the most extreme data point that is no more than 1.5 times the interquartile range from the edge of the box) with n microtissues over 2 independent experiments (n is indicated on top of the box plots) superimposed with a dot plot of the data distribution. ****P < 0.0001, **P < 0.01, *P < 0.05 and n.s. stands for non-significant (i.e. P > 0.05). Statistical significances were determined by two-way analysis of variance (ANOVA) corrected for multiple comparisons using Tukey test. Scale bars are 100 µm. Source data are provided as a Source Data file.

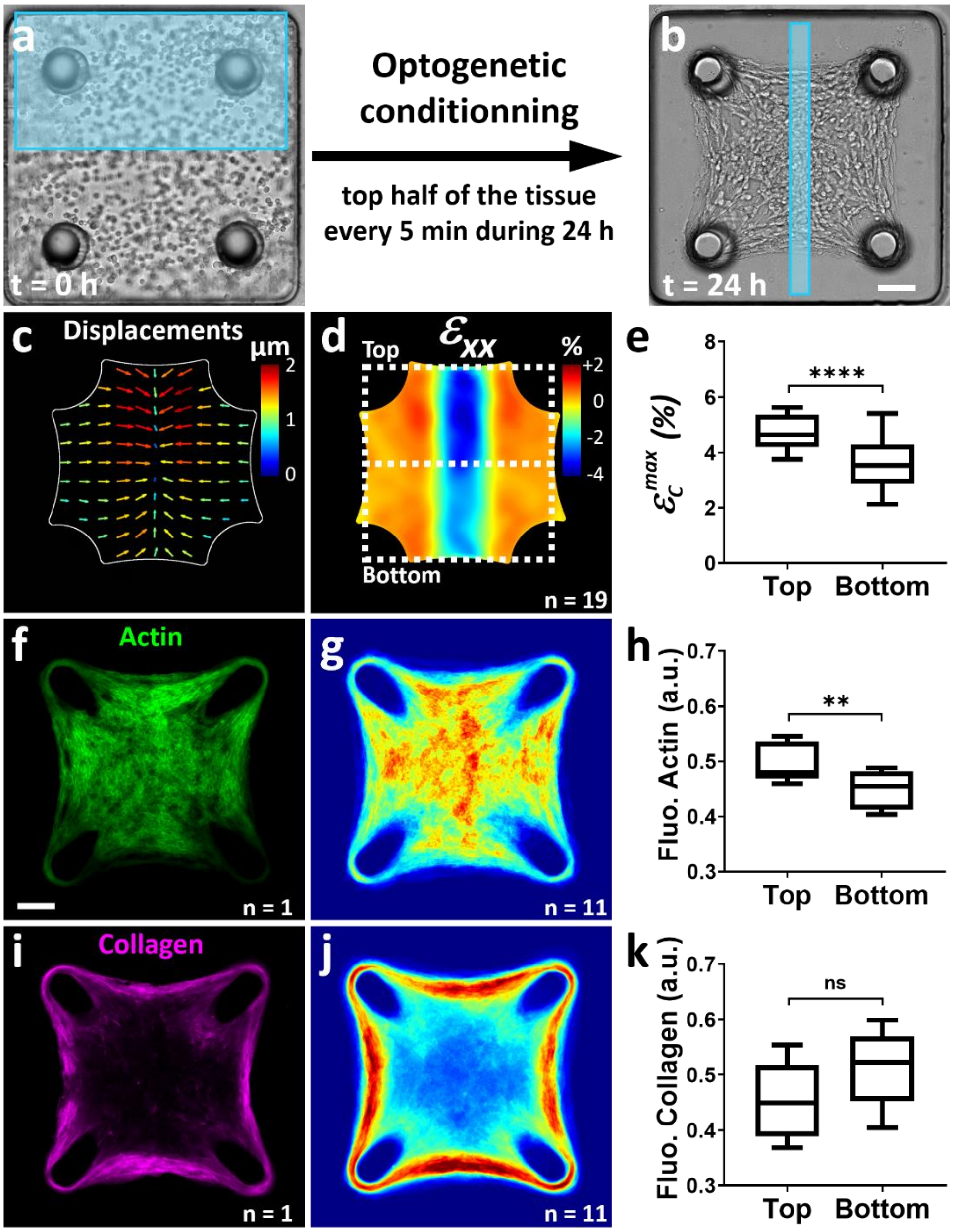

Supp. Figure 7. Optogenetic conditioning of microtissue contractility and architecture. (a) Representative square microtissue at t = 0 h. During the first 24 h of formation, the top half of the microtissue is stimulated every 5 min with a pulse of light (represented by the blue rectangle). (b) After 24 h of formation, a centered, rectangular area of the microtissue (in blue) is stimulated by a single light pulse. Resulting average displacement filed (c), ε_{xx} strain field (d) and comparison of the maximum compression ε_C^{max} in the top, conditioned area and the bottom, non-conditioned area (e). Data are presented as Tukey box plots (i.e. the box extends from the 25th to 75th percentiles, the median is plotted as a line inside the box and the whiskers extend to the most extreme data point that is no more than 1.5 times the interquartile range from the edge of the box) with n = 19 microtissues over 2 independent experiments. **** $P < 0.0001$ determined by two-tailed t-test. Individual (f) and average (g) confocal projection of the fluorescent staining of actin. (h) Corresponding quantification of the fluorescence intensity of the actin staining in the top and bottom area. Individual (i) and average (j) confocal projection of the fluorescent staining of collagen. (k) Corresponding quantification of the fluorescence intensity of the collagen staining in the top and bottom area. Data of actin and collagen fluorescence are presented as Tukey box plots (i.e. the box extends from the 25th to 75th percentiles, the median is plotted as a line inside the box and the whiskers extend to the most extreme data point that is no more than 1.5 times the interquartile range from the edge of the box) with n = 11 microtissues over 2 independent experiments. **$P < 0.01$ and n.s. stands for non-significant (i.e. $p > 0.05$), determined by two-tailed t-test. Scale bars are 100 µm. Source data are provided as a Source Data file.

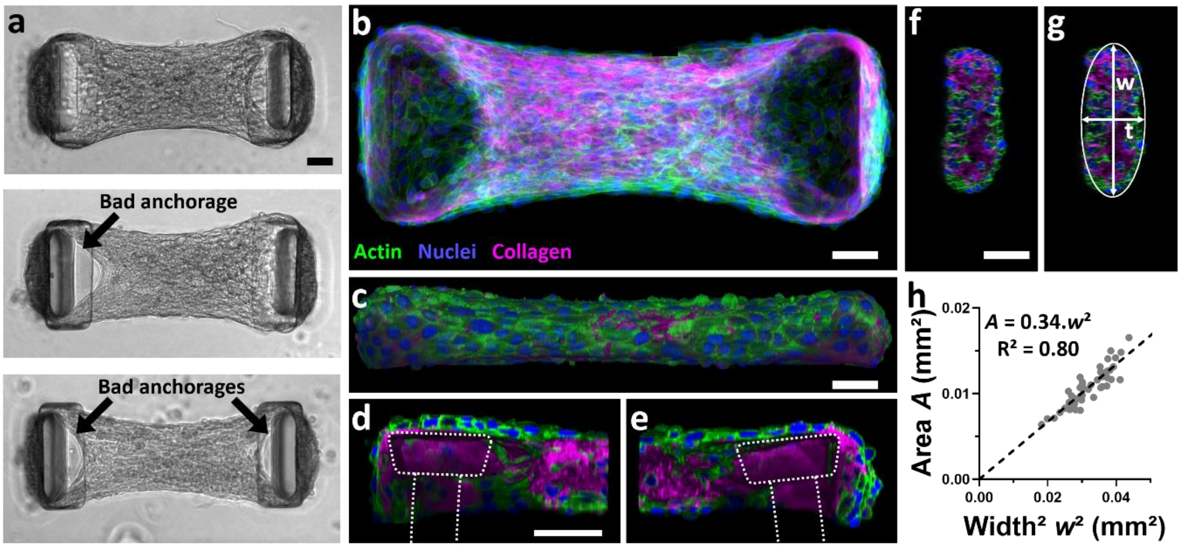

Supp. Figure 8. Anchorage of microtissues to cantilevers and measurement of the cross-section. (a) Representative microtissues properly anchored (top image), with cells wrapping completely the cap of both cantilevers, and poorly anchored to either one (middle image) or two (bottom image) of the cantilevers, with no cells on top of the cantilever and a tissue in a different focal plane than the cantilevers. Poorly anchored microtissues were not selected for experiments. (b) Representative top-view confocal projection of a microtissue stained for actin (green), nuclei (blue) and collagen (magenta). (c) Corresponding orthogonal view in the xz plane. Magnified orthogonal views of the left (d) and right (e) anchorages where cantilevers are outlined with dashed lines. (f) Orthogonal view in the yz plane for measuring tissue cross-section to derive tissue stress σ_{xx}. (g) The yz cross-section can be fitted with an ellipse of width *w* and thickness *t*. (h) The ratio of the cross-sectional area to the squared width w^2 was used to infer cross-sectional area ($A = 0.34w^2$, $R^2 = 0.80$, n = 45 microtissues over 7 independent experiments) from top view images when microtissues could not be fixed immediately after experiment. Scale bars are 50µm. Source data are provided as a Source Data file.

References

1. Walker, M., Godin, M., Harden, J. L. & Pelling, A. E. Time dependent stress relaxation and recovery in mechanically strained 3D microtissues. *APL Bioeng.* **4**, 036107 (2020).
2. Zhao, R., Chen, C. S. & Reich, D. H. Force-driven evolution of mesoscale structure in engineered 3D microtissues and the modulation of tissue stiffening. *Biomaterials* **35**, 5056–5064 (2014).
