# Supplementary material for: Light-driven biological actuators to probe the rheology of 3D microtissues
Source: Nat Commun. 2023 Feb 9;14:717. doi: 10.1038/s41467-023-36371-w (PMC9911700; doi:10.1038/s41467-023-36371-w)
Supplement: Supplementary file 3 — Description of additional Supplementary File [file 41467_2023_36371_MOESM3_ESM.pdf]

Descriptions of additional supplementary files

Supp. Movie 1. Light-controlled local contraction of microtissues. Time lapse of a representative opto-microtissue whose left- then right-half are stimulated with blue light (represented by a blue rectangle) at t = 40 s and t = 21 min, respectively. Upon blue light illumination, the microtissue contracts locally before slowly relaxing, as shown by the PIV-tracking of the displacements.

Supp. Movie 2. Anisotropic contraction of an opto-microtissue upon an isotropic stimulation. Temporal evolution of a representative optomicrotissue and the corresponding strain fields upon the stimulation in its center with a 50 µm diameter discoidal light pattern (represented by a blue disc) at t = 1'20". Despite an isotropic stimulation, the resulting strain is dominated by its x-axis component, as shown by the temporal evolution of εxx and εyy.

Supp. Movie 3. Confocal reconstruction of an opto-microtissue. Z-stack (top) and 3D view (bottom) of a representative opto-microtissue stained for actin (in green), collagen (in magenta) and nuclei (in blue), highlighting the anisotropic orientation of actin and collagen fibers along the xaxis. Cantilevers are reconstructed from thresholded brightfield images.

Supp. Movie 4. Light-induced local contractions evidence the viscoelastic properties of microtissues. Temporal evolution of a representative optomicrotissue, displacement field and εxx strain field upon the stimulation of its left-half with blue light (represented by a blue rectangle) at t = 2'30". The stimulated half is strongly compressed, with a maximum at t = 7'30", while the non-stimulated half is stretched, with a maximum at t = 9'30"

Supp. Movie 5. Contraction is proportional to the area of stimulation. Temporal evolution of a representative opto-microtissue, displacement field and εxx strain field upon the illumination of its center by a 20 µm, a 50 µm and a 100 µm wide stimulation (represented by blue rectangles) at t = 2 min, t = 22 min and t = 42 min, respectively.

Supp. Movie 6. Isotropic contraction of the center of an opto-microtissue. Temporal evolution of a representative opto-microtissue, displacement field and strain fields upon the stimulation in its center with a 200 µm diameter discoidal light pattern (represented by a blue disc) at t = 40 s. The resulting contraction is isotropic, as shown by the displacement field and the similar amplitudes of εxx and εyy

Supp. Movie 7. Anisotropic contraction of the side of an opto-microtissue. Temporal evolution of a representative opto-microtissue, displacement field and strain fields upon the stimulation on its left side with a 200 µm diameter discoidal light pattern (represented by a blue disc) at t = 40 s. The resulting

contraction is anisotropic, as shown by the displacement field and the differences in amplitude between εxx and εyy.

Supp. Movie 8. Confocal reconstruction of a square opto-microtissue. Merged Z-stack (top left) and 3D view (top right) of a representative opto-microtissue stained for actin (in green) and collagen (in magenta). Separated Z-stacks (middle) and magnifications (bottom) of the actin (in green) and the collagen (in magenta) in the left and the center area, highlighting the anisotropic orientation of actin and collagen fibers along the sides, while the center is mostly disorganized. Of note, the intensity of the bottom magnifications is gamma corrected (I_{new} = 255.(I_{old}/255)^γ, with I_{new} and I_{old} the intensity after and before correction, respectively, and γ = 0.6) to better visualize thin, dim collagen fibers despite the intense fluorescence of large collagen bundles.
