# Supplementary material for: Light-driven biological actuators to probe the rheology of 3D microtissues
Source: Nat Commun. 2023 Feb 9;14:717. doi: 10.1038/s41467-023-36371-w (PMC9911700; doi:10.1038/s41467-023-36371-w)
Supplement: Supplementary file 12 — Reporting Summary [file 41467_2023_36371_MOESM12_ESM.pdf]

nature portfolio

Corresponding author(s): Thomas Boudou

Last updated by author(s): Jan 17, 2023

Reporting Summary

Nature Portfolio wishes to improve the reproducibility of the work that we publish. This form provides structure for consistency and transparency in reporting. For further information on Nature Portfolio policies, see our Editorial Policies and the Editorial Policy Checklist.

Statistics

For all statistical analyses, confirm that the following items are present in the figure legend, table legend, main text, or Methods section.

n/a	Confirmed	
☐	☒	The exact sample size (*n*) for each experimental group/condition, given as a discrete number and unit of measurement
☐	☒	A statement on whether measurements were taken from distinct samples or whether the same sample was measured repeatedly
☐	☒	The statistical test(s) used AND whether they are one- or two-sided<br>*Only common tests should be described solely by name; describe more complex techniques in the Methods section.*
☒	☐	A description of all covariates tested
☐	☒	A description of any assumptions or corrections, such as tests of normality and adjustment for multiple comparisons
☐	☒	A full description of the statistical parameters including central tendency (e.g. means) or other basic estimates (e.g. regression coefficient) AND variation (e.g. standard deviation) or associated estimates of uncertainty (e.g. confidence intervals)
☐	☒	For null hypothesis testing, the test statistic (e.g. *F*, *t*, *r*) with confidence intervals, effect sizes, degrees of freedom and *P* value noted<br>*Give P values as exact values whenever suitable.*
☒	☐	For Bayesian analysis, information on the choice of priors and Markov chain Monte Carlo settings
☒	☐	For hierarchical and complex designs, identification of the appropriate level for tests and full reporting of outcomes
☒	☐	Estimates of effect sizes (e.g. Cohen's *d*, Pearson's *r*), indicating how they were calculated

Our web collection on statistics for biologists contains articles on many of the points above.

Software and code

Policy information about availability of computer code

Data collection	Optogenetics stimulation and brightfield imaging were performed using an inverted Nikon Eclipse TI-2 microscope and the software NIS-Elements v.5.20.01<br>Confocal images were obtained with a Leica laser scanning microscope (LSM SP8, Leica) and the software Leica Application Suite X (LAS X) v.3.5.7.
Data analysis	Actin and collagen orientations were evaluated with the Orientation-J plug-in v2.0.5 (http://bigwww.epfl.ch/demo/orientationj/) in Image J. v.1.53m (https://imagej.nih.gov/ij/)<br>The strain in the microtissue was derived from the displacement field determined from the brightfield images using a particle image velocimetry (PIV) algorithm implemented as the toolbox PIVLab v. 2.50 (https://pivlab.blogspot.com/) in MatLab v.2018a<br>The displacement of the top of the cantilevers was tracked using custom MATLAB script (https://github.com/Orion38/Pillar-tracker) and the github web-link is provied in the code availability section of the manuscript.<br>Statistical significances were assessed with Prism v.8.3.0(GraphPad).

For manuscripts utilizing custom algorithms or software that are central to the research but not yet described in published literature, software must be made available to editors and reviewers. We strongly encourage code deposition in a community repository (e.g. GitHub). See the Nature Portfolio guidelines for submitting code & software for further information.

Data

Policy information about availability of data

All manuscripts must include a data availability statement. This statement should provide the following information, where applicable:

- Accession codes, unique identifiers, or web links for publicly available datasets
- A description of any restrictions on data availability
- For clinical datasets or third party data, please ensure that the statement adheres to our policy

Source data are provided as a Source Data file. Because of the large file size, the datasets generated and/or analyzed during the current study are available from the corresponding author on request. A response will be provided in less than 2 weeks.

Human research participants

Policy information about studies involving human research participants and Sex and Gender in Research.

Reporting on sex and gender	n.a.
Population characteristics	n.a.
Recruitment	n.a.
Ethics oversight	n.a.

Note that full information on the approval of the study protocol must also be provided in the manuscript.

Field-specific reporting

Please select the one below that is the best fit for your research. If you are not sure, read the appropriate sections before making your selection.

☒ Life sciences ☐ Behavioural & social sciences ☐ Ecological, evolutionary & environmental sciences

For a reference copy of the document with all sections, see nature.com/documents/nr-reporting-summary-flat.pdf

Life sciences study design

All studies must disclose on these points even when the disclosure is negative.

Sample size	No statistical method was used to predetermine the sample size. Sample sizes were limited by practicality and throughput, and are consistent with previously published similar works in the field, empirically known to provide robust results on microtissue quantifications (Legant et al. PNAS, 2009; Zhao et al., Biomaterials 2014, Hinson et al., Science 2015).
Data exclusions	Only microtissues that were uniformly anchored to the tips of the cantilevers were included in the analysis. Microtissues attached to the bottom of one or both cantilevers were excluded from analysis.
Replication	Experiments have been repeated 2 to 7 times by the same or different experimenters with reproducible outcomes. The number of reproductions are explicited in the figure legends.
Randomization	Samples were randomly allocated into experimental groups
Blinding	Investigators were not blinded to group allocation, as is standard practice in the field.

Reporting for specific materials, systems and methods

We require information from authors about some types of materials, experimental systems and methods used in many studies. Here, indicate whether each material, system or method listed is relevant to your study. If you are not sure if a list item applies to your research, read the appropriate section before selecting a response.

Materials & experimental systems

n/a	Involved in the study
☐	☒ Antibodies
☐	☒ Eukaryotic cell lines
☒	☐ Palaeontology and archaeology
☒	☐ Animals and other organisms
☒	☐ Clinical data
☒	☐ Dual use research of concern

Methods

n/a	Involved in the study
☒	☐ ChIP-seq
☒	☐ Flow cytometry
☒	☐ MRI-based neuroimaging

Antibodies

Antibodies used

Monoclonal Anti-Collagen, Type I antibody produced in mouse, clone COL-1 (Sigma-Aldrich C2456) diluted 1:200 in Tris-buffered saline (TBS, Sigma).
Monoclonal Anti-α-Smooth Muscle Actin produced in mouse, clone 1A4 (Sigma-Aldrich A2547) diluted 1:200 in Tris-buffered saline (TBS, Sigma).
Goat anti-Mouse IgG (H+L) Cross-Adsorbed Secondary Antibody, Alexa Fluo 647 (Invitrogen A-21235) diluted 1:200 in Tris-buffered saline (TBS, Sigma).

Validation

The antibodies were validated by the manufacturers.
List of peer reviewed articles using the anti-collagen antibody: https://www.sigmaaldrich.com/FR/fr/product/sigma/c2456
List of peer reviewed articles using the α-Smooth Muscle Actin antibody: https://www.sigmaaldrich.com/FR/fr/product/sigma/a2547
List of peer reviewed articles using the Goat anti-Mouse antibody: https://www.thermofisher.com/antibody/product/Goat-anti-Mouse-IgG-H-L-Cross-Adsorbed-Secondary-Antibody-Polyclonal/A-21235

Eukaryotic cell lines

Policy information about cell lines and Sex and Gender in Research

Cell line source(s)

Stable opto-RhoA fibroblast cell lines were obtained by viral infection of ARHGEF11-CRY2PHR-mCherry (AddGene #89481) and CIBN-GFP-CAAX (AddGene #79574) in NIH 3T3 fibroblasts from ATCC (#CRL-1658, ATCC).

Authentication

The cell line was authenticated by the manufacturer ATCC. The authors checked the morphology of the cells by microscopy and compared it to ATCC's data (https://www.atcc.org/products/crl-1658). Cells were subcultured thrice a week at 80% confluence or less and cultured for maximum 15 passages, to ensure reproducible phenotype.

Mycoplasma contamination

Cells were regularly tested for mycoplasma and free of contamination.

Commonly misidentified lines
(See ICLAC register)

No commonly misidentified cell lines were used in the study.
